# Education, Income, and Employment and Prevalence of Chronic Disease Among American Indian/Alaska Native Elders

**DOI:** 10.5888/pcd15.170387

**Published:** 2018-03-22

**Authors:** Collette Adamsen, Shawnda Schroeder, Steven LeMire, Paula Carter

**Affiliations:** 1 The University of North Dakota, Center for Rural Health, School of Medicine & Health Sciences, Grand Forks, North Dakota; 2 The University of North Dakota, College of Education & Human Development, Grand Forks, North Dakota

## Abstract

**Introduction:**

Chronic disease studies have omitted analyses of the American Indian/Alaska Native (AI/AN) population, relied on small samples of AI/ANs, or focused on a single disease among AI/ANs. We measured the influence of income, employment status, and education level on the prevalence of chronic disease among 14,632 AI/AN elders from 2011 through 2014.

**Methods:**

We conducted a national survey of AI/AN elders (≥55 y) to identify health and social needs. Using these data, we computed cross-tabulations for each independent variable (annual personal income, employment status, education level), 2 covariates (age, sex), and presence of any chronic disease. We also compared differences in values and used a binary logistic regression model to control for age and sex.

**Results:**

Most AI/AN elders (89.7%) had been diagnosed with at least one chronic disease. AI/AN elders were also more than twice as likely to have diabetes and more likely to have arthritis. AI/AN elders with middle-to-low income levels and who were unemployed were more likely to have a chronic disease than were high-income and employed AI/AN elders.

**Conclusion:**

Addressing disparities in chronic disease prevalence requires focus on more than access to and cost of health care. Economic development and job creation for all age cohorts in tribal communities may decrease the prevalence of long-term chronic diseases and may improve the financial status of the tribe. An opportunity exists to address health disparities through social and economic equity among tribal populations.

## Introduction

Chronic diseases are the leading causes of death and disability in the United States, and nearly half of adults are diagnosed with one or more chronic conditions (1). Many of these conditions are disproportionally prevalent among American Indian/Alaska Natives (AI/ANs) (2,3). The prevalence of chronic conditions among AI/ANs results in low life expectancy (4,5), and AI/ANs are more likely than all other races in the United States to die of heart disease, diabetes, chronic lower respiratory disease, cirrhosis, stroke, pneumonia, kidney disease, and hypertension (4,6).

AI/ANs are more likely than their peers to be at risk for chronic disease as a result of income, education level, employment status, and health behaviors (7,8). One in 4 AI/ANs live in poverty, and tribal communities report the lowest employment rate nationally. The median annual personal income for AI/ANs is far below the national average (7,8). During 2001 and 2002, AI/AN elders (aged ≥55 y) were at greater risk for chronic disease than their non-Hispanic white peers when social status and health behaviors were examined (9). However, the study with these findings relied on a small sample (3,5), was conducted before the Patient Protection and Affordable Care Act and the US recession, and did not explore subgroups in the AI/AN elder population. Other national surveys on the prevalence and risk factors of chronic disease had a small sample of AI/ANs, were disease-specific, omitted analyses of the AI/AN population, or had cell sizes that were too small to report (9–11).

The objective of this national analysis was to identify which social factors, if any, influence the prevalence of chronic diseases among AI/AN elders (aged ≥55 y). We assessed the association of self-reported income, employment status, and education level on the prevalence of chronic disease among 14,632 AI/AN elders from 2011 through 2014.

## Methods

The US Department of Health and Human Services’ Administration for Community Living funds the National Resource Center on Native American Aging (NRCNAA). Data used in this study were taken from the NRCNAA’s 2011–2014 Survey of Elders, which has been administered every 3 years since 2001.

### Survey

NRCNAA faculty developed the paper survey to assist tribes, villages, and homesteads nationally in creating a record of the health and social needs of their elders. The results satisfy the requirement for Title VI Nutrition and Caregiving Grant under the Administration for Community Living. Self-reported data are collected every 3 years on general health status; activities of daily living; vision, hearing, and dental care screenings; health care access; tobacco and alcohol use; weight and nutrition; social support and housing; demographic characteristics; and social functioning. Survey measures mirror those of nationally administered questionnaires to allow for comparison to the US population (12). Data for this study were taken from Cycle V, which covers self-reported health status for AI/AN elders from 2011 through 2014. The University of North Dakota’s institutional review board approved the survey and the proposed method of research; the Official Tribal Council for each participating tribe provided approval through a tribal resolution for the study.

The survey is administered via a Scantron form (Scantron Corporation) on which respondents fill in circles that correspond to the most appropriate responses. The survey also has several write-in responses that are input through image reader technology. Surveys are scanned in-house by NRCNAA staff. To build tribal capacity and improve trust among participants, trained members of the tribe administered the paper surveys to participating elders, reading the questions and filling in the corresponding answers on the form. These individuals were not paid by the NRCNAA directly, but many were employees of the Title VI program. The participating elders were allowed to skip questions they were not comfortable answering. Participating tribes returned all completed surveys to the NRCNAA research team.

All survey data are owned by the tribes. The NRCNAA houses the data, but staff report only in aggregate and under tribal approval. Tribal-specific reports are shared with Title VI directors, who then provide the data to members of the community and to community health groups (to include local public health units). NRCNAA staff and faculty (including authors) include both non-Natives and enrolled members of federally recognized tribes.

### Study population

Tribes participating in the Title VI Nutrition and Caregiving Grant were recruited through the Title VI tribal directors, although we invited all tribes to participate. The 2011–2014 survey cycle included tribally affiliated elders aged 55 years or older. Participants represented all US regions, and 262 (of 566) federally recognized tribes. Within each tribe, researchers identified a simple random sample based on the total number of elders enrolled. The prestudy calculation of required sample size was determined by applying a formula to each tribe. The formula was applied to each of the 262 tribes independently to ensure that the sample obtained was representative of that tribe and not an aggregate representation of all participating tribes collectively. This method allowed results that were representative and generalizable to their population to be shared with each tribe and not to all participating tribes.

Individuals were included in the study if they were aged 55 years or older, an enrolled member of a federally recognized tribe, and eligible to accept services under the Title VI Nutrition and Caregiving Grants. The survey had a 68.5% response rate (14,632 of 21,361 respondents).

### Measures

Independent variables were employment status (employed, unemployed/retired); education level (no education or less than a high school diploma, high school graduate, any education beyond high school); and annual personal income (<$15,000 [low]; $15,000–$49,999 [middle]; ≥$50,000 [high]). Income level was listed categorically in the survey instrument. We controlled for age (55–64 y, 65–74 y, ≥75 y) and sex (male/female). The dependent variable was diagnosis of any chronic disease. Participants replied to the question, “Has a doctor ever told you that you had any of the following diseases (please mark all that apply)?” The presence of chronic disease was assessed with 10 conditions: arthritis, congestive heart failure, stroke, asthma, cataracts, high blood pressure, osteoporosis, depression, diabetes, and cancer. We included cataracts in our analysis because literature on the elderly population more commonly refer to this vision impairment as a chronic condition, and the World Health Organization indicates it as a priority eye disease (13,14). Furthermore, diabetes is associated with the development of cataracts.

### Statistical analysis

We used SPSS (IBM Corporation) to compute summary statistics to identify characteristics of the study population. To determine the prevalence of chronic disease, we created a binary variable to include respondents with at least one of the 10 identified chronic conditions and those without. We converted age from a ratio to a categorical variable, a method most commonly used in health research (15), and converted education level from a 0 to 17 scale to a 3-point scale. We determined the percentage of participants with and without chronic conditions within each category of the independent variables. We computed cross-tabulations for each independent variable and presence of any chronic disease and used a binary logistic regression model (*P* ≤ .05). In the regression model, researchers controlled for age (categorically), and sex (male/female).

## Results

Respondents were mostly female (62.3%), unemployed/retired (69.4%), aged 55 to 64 years (40%), and middle income (56.1%) and had completed some education beyond high school (38.7%). Most AI/AN elders (89.7%) had been diagnosed with at least one of 10 chronic diseases; 69.8% had 2 or more chronic conditions, and 45.2% had 3 or more. The most common chronic diseases among AI/AN elders were high blood pressure (58.9%), diabetes (53.9%), and arthritis (47.2%) (Table 1).

**Table 1:** Percentage of American Indian/Alaska Native (AI/AN) Elders With Diagnosed Chronic Disease (N = 14,632), by Demographic Category, Survey of Elders, 2011–2014

Demographic Characteristic	HBP	Diabetes	Arthritis	Cataracts	Depression	Asthma	Osteoporosis	Cancer	CHF	Stroke
**Overall**	58.9	53.9	47.2	22.5	15.0	13.7	10.8	9.0	8.9	7.7
**Sex^a^**										
Male	59.6	52.9	40.0	19.1	11.4	9.4	3.9	10.0	10.1	8.3
Female	58.6	54.6	51.5	24.5	17.1	16.3	14.9	8.3	8.2	7.3
**Age, y^a^**										
55–64	54.5	49.7	43.0	12.5	17.4	14.8	8.7	6.4	5.7	5.5
65–74	61.3	57.3	48.4	24.3	13.9	13.7	10.9	9.4	9	8.2
≥75	62.8	55.9	52.5	36.4	12.5	11.6	13.9	12.6	14.2	10.7
**Annual personal income, $^a^**										
<15,000 (Low)	58.3	57.5	49.6	21.7	16.2	14.6	11.2	7.8	8.7	8.6
15,000–49,999 (Middle)	59.8	53.3	46.6	23.0	14.4	13.5	10.7	9.5	9.3	7.5
≥50,000 (High)	55.3	44.8	37.5	18.6	11.4	11.1	9.5	10.7	6.1	3.6
**Employment status^a^**										
Unemployed or retired	61.9	57.3	51.7	26.6	17.0	14.5	12.5	10.2	10.9	9.5
Employed	53.1	47.1	36.6	13.3	10.4	12.0	6.6	6.4	4.5	3.5
**Education level^a^**										
Less than a high school diploma	62.2	57.1	52.2	27.5	15.4	14.1	10.9	8.7	11.1	9.5
High school graduate	58.8	52.7	45.8	20.5	13.3	11.7	9.7	8.2	8.3	7.4
Education beyond high school	56.6	52.7	44.8	20.7	16.2	15.0	11.6	9.8	8.0	6.7

Abbreviations: CHF, congestive heart failure; HBP, high blood pressure.

a Significant at *P* ≤ .05.

The prevalence of having one or more chronic diseases was significantly higher among female (91.1%) than male (87.5%) elders; among elders aged 65 to 74 years and aged 75 years or older than those aged 55 to 64 years; among low-income and middle-income elders than high-income elders; among unemployed (92.4%) than employed (83.9%) elders; and among elders who had not completed high school than those with a high school diploma and those with education beyond high school (all *P* ≤ .05) (Table 2).

**Table 2:** Demographic Characteristics of American Indian/Alaska Native Elders With and Without a Diagnosed Chronic Disease (N = 14,632), Survey of Elders, 2011–2014

Demographic Characteristic	With Chronic Disease (n = 13,123^a^)	No Chronic Disease (n = 1,509^a^)
	% (No.)	
**Sex**		
Male	87.5 (4,768)	12.5 (681)
Female	91.1 (8,212)	8.9 (807)
**Age, y**		
55–64	85.5 (5,003)	14.5 (848)
65–74	91.6 (4,849)	8.4 (445)
≥75	93.8 (3,271)	6.2 (216)
**Annual personal income, $**		
<15,000 (Low)	90.6 (4,392)	9.4 (455)
15,000–49,999 (Middle)	90.0 (6,655)	10.0 (737)
≥50,000 (High)	84.6 (794)	15.4 (144)
**Employment status**		
Employed	83.9 (3,459)	16.1 (666)
Unemployed or retired	92.4 (8,623)	7.6 (712)
**Education level**		
Less than a high school diploma	92.2 (3,787)	7.8 (319)
High school graduate	88.8 (4,192)	11.2 (529)
Education beyond high school	88.7 (4,942)	11.3 (630)

a Subcategorical totals may not sum to values for n because of missing data. Percentages for each subcategory omit missing data.

When we controlled for age and sex, middle-income AI/AN elders were 1.3 times as likely as high-income AI/AN elders to be diagnosed with one or more chronic diseases (Table 3). Employed AI/AN elders were less likely than unemployed AI/AN elders to be diagnosed with one or more chronic diseases. When we controlled for age and sex, educational attainment did not have a significant influence on the likelihood that an AI/AN elder would be diagnosed with at least one chronic disease (Table 3).

**Table 3:** Binary Logistic Regression Models for Presence of Chronic Disease Among American Indian/Alaska Native Elders (N = 14,632), by Income, Employment Status, and Education Levels, Controlling for Age and Sex, Survey of Elders, 2011–2014

Variable	B (Standard Error)	Wald χ^2^	*P* Value^a^	Exp(B)
**Income, $**				
≥50,000 (High)^b^	—	11.90	.003	—
15,000–49,999 (Middle)	0.261 (0.105)	6.18	.01	1.298
<15,000 (Low)	0.060 (0.117)	0.27	.60	1.062
**Education**				
Education beyond high school^b^	—	7.18	.03	—
High school graduate	−0.088 (0.072)	1.49	.22	0.916
Less than a high school diploma	0.143 (0.087)	2.72	.10	1.154
**Employed **	−0.715 (0.071)	100.29	<.001	0.489
**Age, y**				
≥75^b^	—	49.69	<.001	—
65–74	−0.157 (0.101)	2.44	.12	0.854
55–64	−0.568 (0.098)	33.57	<.001	0.566
**Female sex**	0.449 (0.062)	51.87	<.001	1.566

a *P* values calculated using Wald χ^2^ test.

b Reference group; cells with a dash indicate that the value was not calculated.

## Discussion

AI/AN elders have a higher prevalence of chronic disease than other races in the US population yet are largely overlooked in research and in proposed federal, social, and tribal interventions. We identified the influence of social variables on the health status of AI/AN elders and found that 89.7% of elders surveyed from 2011 through 2014 had at least one chronic disease. Comparatively, the National Council on Aging, using 2015 Medicaid and Medicare data, reported that 80% of older adults of all races had at least one chronic condition (16,17).

High blood pressure, diabetes, and arthritis were the 3 leading chronic conditions for AI/AN elders. The frequency of hypertension among AI/AN elders (58.9%) nearly mirrored the national average (58%) (16,17). However, other chronic conditions among AI/AN elders occurred at double the national average. Specifically, 54% of the AI/AN elders reported diabetes, compared with only 27% of the US population aged 65 years or older. Roughly 31% of all US adults aged 65 or older were diagnosed with arthritis in 2015, compared with 47.2% of AI/AN elders (16,17).

Federal, state, community, and tribal interventions and policies must explore the prevalence of chronic conditions by race, rather than solely examine general prevalence of chronic disease. Data for AI/AN elders and for the general US elder population identify the prevalence of similar chronic diseases, but special attention must be paid to AI/AN elders. The significant disparity among diseases (eg, diabetes) may also indicate that programs designed to reduce the prevalence of that disease among the general US elder population are not effective, are not reaching tribal populations, or both. As public health units become increasingly responsible for the prevention of chronic disease, these data may be used to develop interventions that are population-specific for each chronic condition.

We established the prevalence of chronic disease among AI/AN elders and then identified demographic categories with higher prevalence of chronic disease. As in national trends, female AI/AN elders were significantly more likely than male AI/AN elders to have a chronic condition (18–21). Approximately 91% of AI/AN female elders had at least one chronic disease, compared with 87.5% of AI/AN male elders. As age increased among elders, so did the likelihood of having a chronic condition, which mirrors national trends (21). 

The prevalence of chronic disease among AI/ANs who were employed was significantly lower than for those who were unemployed; similarly, those with high income were less likely than middle-income and low-income elders to have a chronic condition. These trends were also reported by the National Center for Health Statistics for the general US population in 2013 (15). Cost of care is the most common reason patients with chronic conditions delay treatment or prevention (regardless of income or employment status) (22). Therefore, there is heightened concern for the AI/AN elder population, as most were unemployed and middle-income to low-income and less likely to then afford health care services both on and off of the reservation.

Although the Indian Health Service (IHS) provides care at reduced cost to AI/ANs on the reservation, access to that care is limited because IHS consistently is underfunded (23,24). During 2009 and 2010, Indian health expenditures per capita were one-third of the expenditures for Medicare, and they were lower per capita than those for veterans, Medicaid patients, and participants in the Federal Employees Health Benefits Program (25). AI/ANs also have high uninsured rates, making it more difficult to access care outside of IHS, especially for those who are low-income (23). These issues of access to affordable care likely contribute to the increased prevalence of chronic disease among AI/AN elders who are low-income and unemployed.

To prevent chronic diseases and improve the health status of those with chronic diseases, communities and programs can investigate job creation for AI/AN elders. Elders may also require resources like transportation and job training. Economic development and job creation for all age cohorts in tribal communities will benefit long-term chronic disease prevalence and can improve the financial status of the tribe.

Results also indicate a need to identify solutions that are focused more on population health than on access to or cost of care. Communities have developed programs that rely on collaboration between public health units, at-risk tribal populations, and local health care systems to address prevention and early detection services (26–29). As public health units take on a larger responsibility for population health, this data can and is being used to target population subgroups in the AI/AN population who have an increased prevalence of chronic conditions. Tribal public health units can identify subgroups (eg, women) that have a higher prevalence of certain chronic conditions and provide education and prevention that is specific to them (26). Likewise, as AI/ANs seek information, prevention, and treatment outside of IHS, local public health units need to recognize that AI/AN elders that use their services likely have multiple chronic conditions. Shaw et al discussed health literacy and the need for communities and public health units to understand the socioeconomic and cultural differences of at-risk populations (29). Programs exist that effectively incorporate cultural traditions in public and tribal health services (10,27,29,30). These models can improve the health of tribal communities and their elders.

This study has several limitations. First, the national definition of elderly is typically aged 65 or older. We assessed chronic disease among AI/AN elders aged 55 or older. This decision was in response to the lower average life expectancy of AI/ANs compared with that of the general US population. However, doing so made it difficult to compare AI/AN elders’ prevalence of chronic disease to the prevalence of disease among all older people in the United States with any certainty. This limitation, however, provides a conservative measure of chronic disease among AI/ANs elders, recognizing that research indicates a higher prevalence among older cohorts. Omitting respondents aged 55 to 64 years would have resulted in higher prevalence rates for the AI/AN population.

The self-reported diagnosis of a chronic disease may also be problematic. There is risk of both underreporting and overreporting. Elders may not want to identify with a given disease or may have low health literacy and misunderstand or forget a diagnosis. Elders may also self-diagnose and indicate a chronic disease that has not been clinically diagnosed. Finally, the results addressed AI/ANs as one collective people, although AI and AN populations experience vastly different health barriers, practice different traditions, and vary in some health outcomes. The decision to speak to the AI/AN elder population collectively was made to ensure a large enough sample to generalize for tribal populations and to have large enough cell sizes to conduct both a factor analysis and cross-tabulations. Future research may investigate the 2 populations independently to determine whether differences in the prevalence of chronic disease exist. In addition, the variable “employment” previously omitted a distinction between “retired” and “unemployed,” limiting respondents to indicate only “yes, employed full-time”; “yes, employed part-time”; or not employed. Future cycles of the survey will now include “retired” and “unemployed” as separate categories, which will allow for more granularity in the discussion of chronic disease among people who are not employed.

We found a higher overall prevalence of chronic disease among AI/AN elders compared with the older US population and substantially higher rates of both diabetes and arthritis among AI/AN elders. Finally, we found that a significantly higher prevalence of chronic disease exists among AI/AN elders who are unemployed and middle-income or low-income. These findings call for economic and social interventions outside those typically related to access to care. These results may be used to develop public health and community programs and interventions at the tribal level dedicated to improving the health of AI/AN elders, especially those with hypertension, arthritis, or diabetes, and those who are middle- to low-income or unemployed. An opportunity exists to address health disparities through social and economic equity among tribal populations.
